# Laparoscopic and endoscopic cooperative surgery for a giant duodenal Brunner’s gland hamartoma

**DOI:** 10.1055/a-2871-7944

**Published:** 2026-06-01

**Authors:** Wataru Kurihara, Hiroyuki Yamamoto, Masaru Hayami

**Affiliations:** 1 Department of GastroenterologyCancer Institute Hospital of Japanese Foundation for Cancer ResearchTokyoJapan; 2 Department of Gastroenterology and Metabology 38050 Ehime University Graduate School of MedicineEhimeJapan; 3 Department of Gastroenterological SurgeryCancer Institute Hospital of Japanese Foundation for Cancer ResearchTokyoJapan

Duodenal laparoscopic and endoscopic cooperative surgery (D-LECS) is effective in preventing delayed perforation by reinforcing the duodenal wall with laparoscopic seromuscular suturing 1 2 , as well as removing large duodenal lesions 3 4 . Laparoscopic straightening of the stomach and duodenum can also improve endoscopic visualization and maneuverability for lesions located on the duodenal flexure points, facilitating safer resection.

**Fig. 1:**
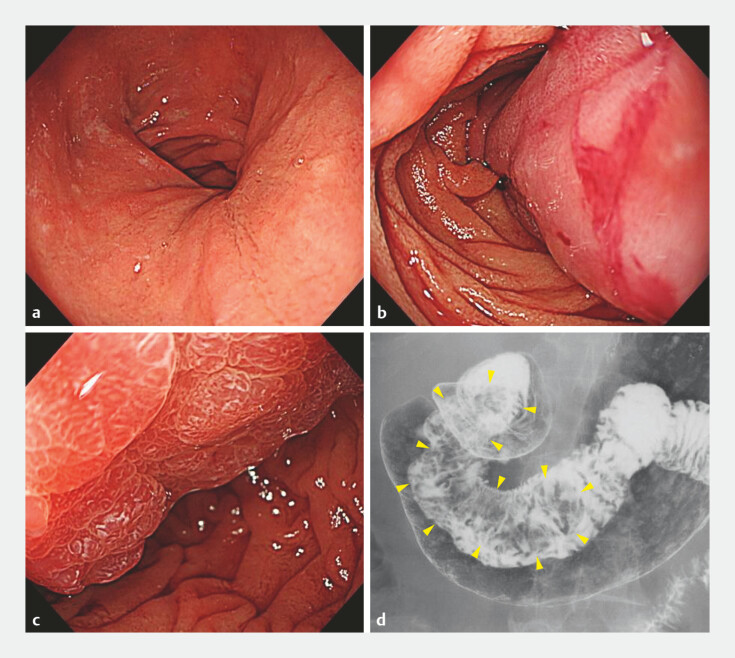
**a** The base of the large duodenal lesion is located on the posterior wall of the superior duodenal angle, and its weight has caused traction on the mucosa. **b** The lesion is pedunculated and extends horizontally, occupying the lumen. **c** Hyperplastic changes are present in portions of the lesion surface, and histopathological examination of a biopsy specimen suggests a hyperplastic polyp. **d** An upper gastrointestinal series shows a 60-mm radiolucent area.

**Fig. 2:**
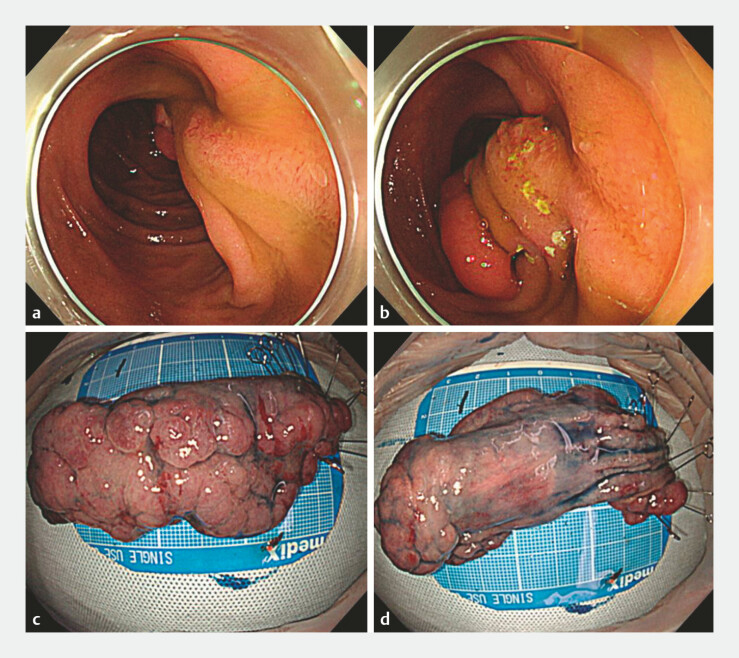
**a** and **b** During duodenal laparoscopic and endoscopic cooperative surgery, laparoscopic straightening of the stomach and duodenum improved endoscopic visualization and maneuverability, facilitating accurate marking at the base of the lesion. **c** and **d** Histopathological examination reveals a 63 × 33-mm Brunner’s gland hamartoma with negative resection margins.

Herein, we present a case of a giant duodenal lesion that we recently resected via D-LECS. The patient was a woman in her 70s who underwent esophagogastroduodenoscopy for anemia, which revealed a pedunculated lesion extending from the superior duodenal angle to the horizontal portion ( Fig. 1 **a–c** ). An upper gastrointestinal series showed a 60-mm radiolucent area ( Fig. 1 **d** ). Although histopathological examination of a biopsy specimen suggested a hyperplastic polyp, we suspected the duodenal Brunner’s gland hamartoma based on experience 5 . Because the lesion was large and located on the superior duodenal angle flexure, endoscopic submucosal dissection alone was expected to be difficult owing to poor maneuverability and oral retrieval issues. Therefore, following a discussion between endoscopists and surgeons, D-LECS was selected ( Video 1 ). This approach improved the operative field ( Fig. 2 **a** and **b** ), facilitating en bloc resection using a DualKnife (Olympus, Tokyo, Japan), an ITknife nano (Olympus), and a traction device. Because the specimen could not pass through the esophagogastric junction, the gastric wall was opened laparoscopically for retrieval through the incision. The resection site was reinforced with laparoscopic seromuscular suturing and endoscopic clip closure to prevent delayed perforation and postoperative bleeding. The total operative time was 325 minutes, including 41 minutes for endoscopic submucosal dissection. No adverse events occurred. Histopathological examination revealed a 63 × 33-mm Brunner’s gland hamartoma with negative resection margins ( Fig. 2 **c** and **d** ). To our knowledge, no previous report has described D-LECS for a giant duodenal Brunner’s gland hamartomas. This case highlights the feasibility of this approach.

Laparoscopic and endoscopic cooperative surgery for a giant duodenal Brunner’s gland hamartoma.Video 1

Endoscopy_UCTN_Code_CCL_1AB_2AD_3AF Endoscopy_UCTN_Code_TTT_1AO_2AG_3AF
